# Acute and chronic glucocorticoid treatments regulate astrocyte-enriched mRNAs in multiple brain regions *in vivo*

**DOI:** 10.3389/fnins.2013.00139

**Published:** 2013-08-07

**Authors:** Bradley S. Carter, David E. Hamilton, Robert C. Thompson

**Affiliations:** ^1^Neuroscience Graduate Program, University of MichiganAnn Arbor, MI, USA; ^2^Molecular and Behavioral Neuroscience Institute, University of MichiganAnn Arbor, MI, USA; ^3^Department of Psychiatry, University of MichiganAnn Arbor, MI, USA

**Keywords:** glucocorticoids, corticosterone, RNA, messenger, brain, astrocytes, mice

## Abstract

Previous studies have primarily interpreted gene expression regulation by glucocorticoids in the brain in terms of impact on neurons; however, less is known about the corresponding impact of glucocorticoids on glia and specifically astrocytes *in vivo*. Recent microarray experiments have identified glucocorticoid-sensitive mRNAs in primary astrocyte cell culture, including a number of mRNAs that have reported astrocyte-enriched expression patterns relative to other brain cell types. Here, we have tested whether elevations of glucocorticoids regulate a subset of these mRNAs *in vivo* following acute and chronic corticosterone exposure in adult mice. Acute corticosterone exposure was achieved by a single injection of 10 mg/kg corticosterone, and tissue samples were harvested 2 h post-injection. Chronic corticosterone exposure was achieved by administering 10 mg/mL corticosterone via drinking water for 2 weeks. Gene expression was then assessed in two brain regions associated with glucocorticoid action (prefrontal cortex and hippocampus) by qPCR and by *in situ* hybridization. The majority of measured mRNAs regulated by glucocorticoids in astrocytes *in vitro* were similarly regulated by acute and/or chronic glucocorticoid exposure *in vivo*. In addition, the expression levels for mRNAs regulated in at least one corticosterone exposure condition (acute/chronic) demonstrated moderate positive correlation between the two conditions by brain region. *In situ* hybridization analyses suggest that select mRNAs are regulated by chronic corticosterone exposure specifically in astroctyes based on (1) similar general expression patterns between corticosterone-treated and vehicle-treated animals and (2) similar expression patterns to the pan-astrocyte marker Aldh1l1. Our findings demonstrate that glucocorticoids regulate astrocyte-enriched mRNAs *in vivo* and suggest that glucocorticoids regulate gene expression in the brain in a cell type-dependent fashion.

## Introduction

At the level of the organism, stress occurs via environmental or psychological stimuli (stressors) that disrupt homeostasis. The classic stress response involves communication between the brain and the rest of the body through the hypothalamic-pituitary-adrenal (HPA) axis. In response to stress, the brain integrates signals through the brainstem and the limbic system (e.g., prefrontal cortex, hippocampus, and amygdala), which in turn activates signaling cascades through the hypothalamus and pituitary gland that result in the stimulation of the adrenal glands and release of glucocorticoids into the circulatory system. Glucocorticoids are the major output hormones of the HPA axis and enable the organism to adapt to stressors by influencing many physiological pathways (e.g., modulate attention and appraisal, alter energy metabolism, regulate immune signaling, modulate memory formation, etc.) (De Kloet et al., 2005; Lupien et al., 2009). Glucocorticoids also act in negative feedback mechanisms at multiple levels of the HPA axis (e.g., limbic system, hypothalamus) to regulate stress response activation and return the system to baseline following stress. Appropriate stress response is thus critical for appropriate responsiveness to external stimuli. Chronic disruption of appropriate stress signaling (e.g., altered HPA axis signaling, chronic stress) can have drastic impact on well-being and has been linked to numerous disease states, including Cushing's disease [HPA hyperactivity (Newell-Price et al., 2006)], Addison's disease [HPA hypoactivity (Ten et al., 2001)], and multiple psychiatric disorders, including depression and bipolar disorder (McEwen, 2005). Given the importance of the stress response to health and disease, there is great interest in understanding the impact of glucocorticoids on the brain, both in terms of acute exposure (e.g., stress mechanisms) and chronic exposure (e.g., disease states).

At the cellular level, glucocorticoids are known to act as transcriptional regulators through interaction with their associated receptors (i.e., mineralocorticoid receptor, glucocorticoid receptor) (Strachan and Read, 1999). These receptors have unique neuroanatomical expression patterns but are both prominently expressed in limbic structures (e.g., prefrontal cortex, hippocampus) to facilitate HPA axis signaling. Glucocorticoids readily pass through the cell membrane and bind their inactive receptors that are complexed with chaperone proteins in the cytoplasm. Ligand-bound receptors are activated and translocate to the nucleus [mechanisms reviewed in Vandevyver et al. (2012)]. Activated receptors can bind to DNA sequences known as glucocorticoid response elements (GREs) and upregulate or downregulate target gene expression. Glucocorticoids are known to regulate many mRNAs in the brain. While numerous studies have reported *in vitro* findings of glucocorticoid regulation on a gene by gene basis, fewer studies have looked at glucocorticoid regulation in the brain at the level of the transcriptome *in vivo*. A limited number of gene expression profiling studies of glucocorticoid mRNA regulation using brain tissue have found that numerous mRNAs are regulated by glucocorticoids in hippocampal slices (Datson et al., 2001) and that the observed regulation varies by the duration of glucocorticoid exposure (Morsink et al., 2006).

The brain consists of many diverse cell types (e.g., neurons, astrocytes, oligodendrocytes, microglia) that vary significantly in function. Historically, investigations of the brain have focused on neurons, but there is increasing evidence that glial cells play an active role in numerous brain processes. Astrocytes participate in many functions, including neurotransmission regulation, electrical coupling and gap junctions, gliotransmission, metabolism and the blood brain barrier, and astrogliosis and immune function [reviewed in Maragakis and Rothstein (2006)]. In addition to the growing functional importance of glial cells, all of these cell types are known to express glucocorticoid receptors and are thus likely responsive to glucocorticoid signaling (McEwen et al., 1968; Vielkind et al., 1990; Sierra et al., 2008). Previous studies examining the impact of glucocorticoids upon brain tissues have routinely interpreted the action of these steroids in terms of neuronal physiology, but glucocorticoids are also known to impact glial cells. For example, glucocorticoids can inhibit astrocyte proliferation *in vivo* (Unemura et al., 2012). In terms of mRNA regulation, glucocorticoids have been shown to regulate a limited number of astrocyte mRNAs, including glutamine synthetase and glial fibrillary acidic protein (GFAP) (Nichols et al., 1990; Laping et al., 1994). Beyond these limited reports, the impact of glucocorticoids on global gene expression in cell types other than neurons in the brain is largely unknown.

There is also a clinical interest in understanding how glucocorticoids impact astrocytes due to their growing association with major depression, a disorder strongly associated with altered HPA axis signaling. Postmortem morphological studies have observed changes in astrocytes associated with depression (e.g., decreased astrocyte density and increased astrocyte size in prefrontal cortex) (Rajkowska et al., 1999; Miguel-Hidalgo et al., 2000), and postmortem brain transcriptional profiling studies have also identified astrocyte-associated mRNAs as being altered in depression (Barley et al., 2009; Bernard et al., 2010). However, how and even if glucocorticoids influence astrocytes in pathological conditions such as depression is currently speculative, and the extent of glucocorticoid regulation occurring in astrocytes *in vivo* under basal conditions is not well-understood. We recently characterized glucocorticoid receptor-mediated regulation of mRNAs in cortical and hippocampal astrocytes *in vitro*, defining glucocorticoid-regulated mRNA targets that included both astrocyte-specific mRNAs and mRNAs ubiquitously expressed among cell types in the brain (Carter et al., 2012). However, the physiological relevance of this glucocorticoid regulation observed in astrocyte cell culture has not been investigated in the intact brain. Here we have assessed whether a subset of mRNAs regulated by glucocorticoids in astrocytes *in vitro* are responsive to glucocorticoids *in vivo* following acute and chronic glucocorticoid exposure, including mRNAs reported to be enriched in astrocytes (Cahoy et al., 2008).

## Materials and methods

### Animal treatments and brain tissue collection

All animal procedures at the University of Michigan were approved by UCUCA (University Committee on Use and Care of Animals) and monitored by ULAM (Unit for Laboratory Animal Medicine). Adult C57B/6 male mice (8 weeks of age) were obtained from Charles River. For all experiments, mice were group-housed (*n* = 3–5 per cage). Mice were housed for 1 week under basal conditions (14:10 light dark cycle) before experiments. To collect tissue samples, mice were euthanized using cervical dislocation and decapitation. For both the acute and chronic glucocorticoid exposure protocols, upon euthanasia, trunk blood was collected, and brains were removed and bisected along the mid-sagittal plane. One brain hemisphere was frozen in chilled isopentane (−35°C) and stored at −80°C for subsequent tissue sectioning and *in situ* hybridization analyses. The remaining brain hemisphere was further dissected on wet ice to collect hippocampi and prefrontal cortex tissues for quantitative PCR (qPCR) analyses. These tissue samples were frozen immediately on dry ice and stored at −80°C for subsequent RNA extraction.

### Acute glucocorticoid mouse model

Mice were injected (s.c.) with 10 mg/kg corticosterone or vehicle (1% ethanol solution) 1 h after lights on. Mice were returned to their home cages for specified time durations prior to euthanasia and sample collection. Corticosterone (Cort, Cat. #C2505) was obtained from Sigma Aldrich.

### Chronic glucocorticoid mouse model

Chronic corticosterone exposure was produced based on previously published protocols (Gourley et al., 2008; Karatsoreos et al., 2010; Lee et al., 2010). Mice were given access to drinking water containing 10 mg/mL corticosterone or vehicle for 2 weeks (*n* = 5/group). Drinking solutions were replaced 3 times over course of 2 weeks (every 4–5 days). On day 14, mice were weighed on an analytical balance. Mice were then euthanized 1 h after lights on, and brains were removed. Adrenal glands, spleen, and thymus tissues were dissected on ice and weighed on an analytical balance.

### Plasma corticosterone assay

Immediately following decapitation, trunk blood was collected and mixed with 0.5 M EDTA (pH 8.0, final concentration in blood sample ~10 μM) and stored immediately on wet ice. Blood samples were subsequently spun in a table-top centrifuge (3500 × g for 10 min at 4°C). Blood plasma was transferred to a fresh 1.5 mL tube and stored at −80°C. Plasma corticosterone concentrations were measured using a corticosterone double antibody radioimmunoassay kit (MP Biomedicals, Cat. #07120102). Individual samples were measured in triplicate, including standard curve controls; the average value was used for subsequent calculations.

### RNA isolation

Total RNA samples were isolated from tissues using Trizol reagent per manufacturer's protocol (Invitrogen), and RNA concentrations were obtained using a Nanodrop spectrophotometer (Thermo Scientific).

### Quantitative PCR (qPCR) analysis

RNA samples (500 ng–1 μg) were converted to cDNA using Superscript II via random hexamer priming (Invitrogen). Approximately Fifty percentage of each cDNA reaction was used for Applied Biosystems (ABI) Taqman mRNA qPCR assays in custom Low-Density Array (TLDA) format (ABI #4346799) with Taqman reagents (ABI #4440048). The Taqman arrays were processed on an Applied Biosystems Viia7 instrument according to manufacturer protocols. mRNAs were selected for measurement based on combinations of the following of factors as defined previously (Carter et al., 2012); (1) glucocorticoid sensitivity vs. insensitivity in primary astrocyte cultures, (2) magnitude of glucocorticoid regulation, (3) previous reporting of glucocorticoid sensitivity vs. novel glucocorticoid regulation, and (4) previous association with astrocyte function and/or astrocyte-enrichment (Cahoy et al., 2008). Previously reported glucocorticoid regulation vs. novel glucocorticoid regulation was determined by literature analysis of each individual gene (i.e., using PubMed and Google Scholar search tools, terms used: gene symbol and/or probe ID + “glucocorticoids, corticosteroids, corticosterone, dexamethasone, prednisone”). mRNA expression was measured in technical triplicate per sample. Taqman mRNA assays were selected based on manufacturer recommendations; all but one assay spanned exon-exon junctions (Glul). Specific Taqman mRNA assays used in this analysis are: Actb (Mm01205647_g1), Adora2b (Mm00839292_m1), Aldh1l1 (Mm00550947_m1), Atp6v1b2 (Mm00431987_m1), Ch25h (Mm00515486_s1), Egr2 (Mm00456650_m1), Fgfr1 (Mm00438930_m1), Fgfr3 (Mm00433294_m1), Fkbp5 (Mm00487401_m1), Folh1 (Mm00489655_m1), Foxo1 (Mm00490672_m1), Gap43 (Mm00500404_m1), Gfap (Mm01253033_m1), Gja1 (Mm00439105_m1), Gjb6 (Mm01317508_m1), Glul (Mm00725701_s1), Hdac7 (Mm00469520_m1), Klf9 (Mm00495172_m1), Mapk4 (Mm00554001_m1), Mertk (Mm00434920_m1), Pdk4 (Mm01166879_m1), Per1 (Mm00501813_m1), Phlda1 (Mm00456345_g1), Prodh (Mm00448401_m1), Sgk1 (Mm00441380_m1), Slc1a2 (Mm00441457_m1), Slc1a3 (Mm00600697_m1), Sult1a1 (Mm01132072_m1), Syn2 (Mm00449780_m1), Txnip (Mm00452393_m1), Wnt7a (Mm00437354_m1). Gene symbols, definitions, and reported astrocyte fold-enrichment (Cahoy et al., 2008) for measured mRNAs are listed in Table 1.

**Table 1:** **mRNAs assessed for glucocorticoid regulation in the brain *in vivo***.

**Symbol**	**Gene (protein)**	Astrocyte fold-enrichment^*^
Adora2b	Adenosine A2b receptor	17.4
Aldh1l1	Aldehyde dehydrogenase 1 family, member L1	54.0
Atp6v1b2	V-ATPase B2 subunit	–
Ch25h	Cholesterol 25-hydroxylase	–
Egr2	Early growth response protein 2	–
Fgfr1	Fibroblast growth factor receptor 1	8.8
Fgfr3	Fibroblast growth factor receptor 3	27.2
Fkbp5	FK506 binding protein 5	–
Folh1	Folate hydrolase 1	11.4
Foxo1	Forkhead box protein O1	3.8
Gap43	Growth associated protein 43	–
Gfap	Glial fibrillary acidic protein	84.9
Gja1	Gap junction protein, alpha 1 (connexin-43)	20.9
Gjb6	Gap junction protein, beta 6 (connexin-30)	32.7
Glul	Glutamine synthetase	8.5
Hdac7	Histone deacetylase 7	–
Klf9	Kruppel-like factor 9	3.1
Mapk4	Mitogen-activated protein kinase 4	5.6
Mertk	C-mer proto-oncogene tyrosine kinase	33.0
Pdk4	Pyruvate dehydrogenase kinase, isozyme 4	20.8
Per1	Period circadian clock 1	1.9
Phlda1	Pleckstrin homology-like domain family A member 1	–
Prodh	Proline dehydrogenase 1	29.4
Sgk1	Serum/glucocorticoid-regulated kinase 1	–
Slc1a2	Solute carrier family 1, member 2 (EAAT2/GLT-1)	46.7
Slc1a3	Solute carrier family 1, member 3 (EAAT1/GLAST)	43.0
Sult1a1	Sulfotransferase 1A1	12.8
Syn2	Synapsin II	–
Txnip	Thioredoxin-interacting protein	5.3
Wnt7a	Wingless-related MMTV integration site 7A	7.6

* mRNA expression in astrocytes compared to neurons and oligodendrocytes (Cahoy et al., 2008).

### *In situ* hybridization (ISH)

**Figure 1:**
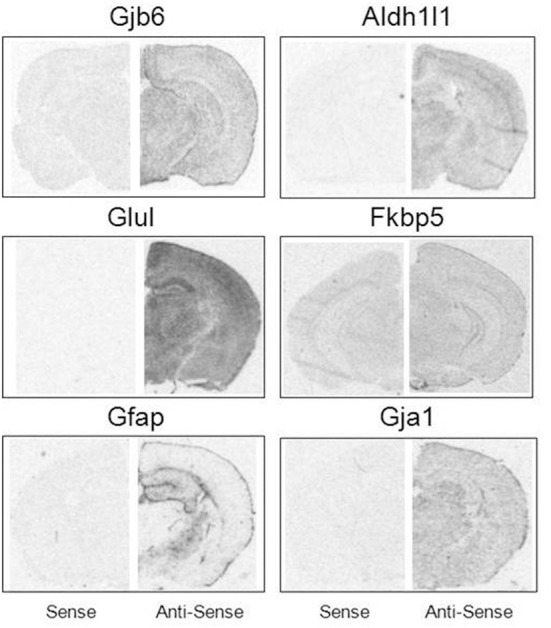
**Specificity of mRNA in situ hybridization probes.** Sense (**Left**) and antisense (**Right**) comparisons of ISH probe signal under matched exposure times per probe.

Brain hemisections were cut on a cryostat (10 μm thick), mounted on Superfrost microscope slides (2 sections per slide), and stored at −80°C prior to ISH experiments. To create ISH probes, specific mRNA domains that displayed low levels of nucleotide homology were amplified by PCR (Native Taq DNA polymerase, Invitrogen) using primers designed with NCBI Primer Blast software (Table 2). PCR amplicons were cloned into pCR-II-TOPO Vector; plasmids were transformed using TOP10 cells (Invitrogen). Plasmid DNA was isolated using a QIAprep Spin Miniprep Kit (Qiagen). The identity of all plasmid clones was verified by Sanger Sequencing (DNA sequencing core, University of Michigan). Plasmids were linearized using appropriate restriction enzymes (NEB). Linearized plasmids were used to create ^35^S-RNA probes using T7 or SP6 RNA polymerases (varied by plasmid). Briefly, linearized plasmid (100–500 ng) was combined with 1 μl each of ATP, GTP, and CTP (10 mM), 1 μl RNAse inhibitor, 1.66 μl DTT (100 μM), 4 μl 5 × transcription buffer (Promega) and 7.8 μl of ^35^S-UTP (12.5 μCi/ul, Perkin Elmer). Probes were purified using BioRad P-6 columns; the effluent was measured with a scintillation counter and then diluted with hybridization solution (~2 × 10^6^ cpms of probe in 40 μl hybridization buffer per slide). All remaining tissue processing steps were performed according to published lab protocols (Carter et al., 2010). Probe hybridization specificity was defined in control experiments by sense probes which failed to yield autoradiographic signals above background. Sense probes demonstrate significantly lower signal compared to antisense probes for all mRNAs assessed (Figure 1).

**Table 2:** **Primers for *in situ* hybridization probes**.

**mRNA**	**Forward primer**	**Reverse primer**
Fkbp5	GGACCACGCTATGGTTTTGG	AACATGTTGGCGTACACCCT
Gja1	AGTGAAAGAGAGGTGCCCAG	TGCCGTGTTCTTCAATCCCA
Gjb6	AAGAACACAGGCGCAGAGAA	TTGTCCAGGTGACTCCAAGG
Glul	CCTGGACCCCAAGGCCCGTA	CGGTTGGCAACACCGGCAGA
Gfap	CTGGCCCAACAGCAGGTCCAC	TCCAGGCTGGTTTCTCGGATCTGG
Aldh1l1	GGGGACAGGAGGGTGCTAAGTC	TGTCATCCCCTGGAACTATCCC

^*^Primers listed 5 ′ ≥ 3 ′.

### Densitometry analysis

Anatomical boundaries of the prefrontal cortex and hippocampus where defined based upon reference to the mouse brain atlas of Paxinos and Franklin (2001). Autoradiograms from ISH were scanned with a ScanMaker 1000XL Pro Flatbed Scanner (Microtek, Carson, CA) using SilverFast Ai Imaging Software (LaserSoft Imaging, Sarasota, FL). The scanned images were analyzed based on optical density (OD) measurements using ImageJ software (Version 1.45S, NIH). For each probe, a normalized OD value for each section image was determined by subtracting a background value from the OD value (Background = background mean + 3.5 * standard deviation of background mean). Background measurements were taken from a non-tissue area of each film. Four section images were measured for each brain region per animal; an average normalized value of the 4 sections per probe was used for downstream analyses.

### Statistical analysis

For qPCR experiments, differential expression analysis was performed using the delta-delta-Ct method [(Livak and Schmittgen, 2001), β-actin as control reference] using Statminer software (Integromics). An average of Ct-values from technical replicates was taken as the Ct-value for each gene measurement; individual Ct-values identified as outliers via the Grubbs' outlier test were excluded from downstream analyses. For comparisons between groups in terms of qPCR analysis of mRNA differential expression, measurements were compared using a moderated *t*-test; statistical significance was defined as *p* < 0.05. The relationship between corticosterone levels over time due to corticosterone injection was analyzed using a Two-Way ANOVA with multiple replicates; comparison of groups at individual time points was performed *post-hoc* using Fisher's least-significant difference (LSD) test. For individual comparisons between groups in terms of organ weight, body weight, corticosterone levels, and ISH analysis, measurements were compared using a two-tailed student's *t*-test; statistical significance was defined as *p* < 0.05. The standard error of the mean (SEM) was used for error bars on graphs.

## Results

### Single corticosterone injection results in transient rise of corticosterone plasma levels

**Figure 2:**
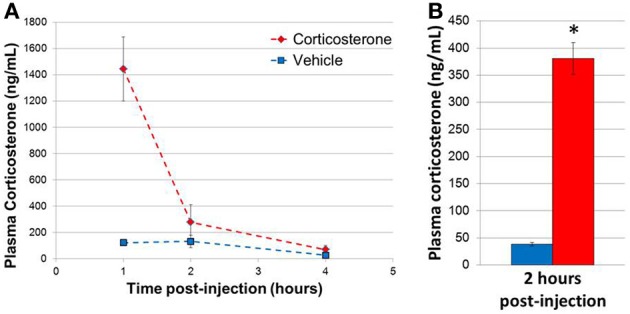
**Single injection of 10 mg/kg corticosterone results in transient rise in plasma corticosterone levels in mice. (A)** Time course of plasma corticosterone levels following injection (*n* = 3/treatment/time point). **(B)** Plasma corticosterone levels in mice (2 h post-injection exposure) used in qPCR experiments (*n* = 8/group). Error bars = SEM. ^*^p < 0.05.

In order to determine the plasma concentrations of corticosterone *in vivo* following a single bolus injection of corticosterone, 3 sets of mice were injected with 10 mg/kg corticosterone or vehicle solution, and blood samples were then collected at 1, 2, and 4 h post-injection (*n* = 3 animals/group/time point, 18 animals total; Figure 2A). Two-Way ANOVA revealed a significant effect of treatment [*F*_(1, 12)_ = 9.54, *p* = 0.009], time point [*F*_(2, 12)_ = 7.08, *p* = 0.009] and a significant interaction between treatment and time point [*F*_(2, 12)_ = 5.51, *p* = 0.02]. *Post-hoc* comparisons revealed a trend for a significant effect of treatment on corticosterone levels at 1 h (Fisher's LSD *p* = 0.06). Overall, corticosterone-treated animals tended to exhibit higher corticosterone levels at each time point compared with vehicle-treated animals, and the difference between treatment groups was dependent on the length of exposure. Specifically, a 10 mg/kg dose of corticosterone increased plasma corticosterone concentration to a supraphysiological level by 1 h. The plasma corticosterone level remained significantly elevated at 2 h at levels similar in magnitude to acute stress (Ma et al., 1997; McClennen et al., 1998) and then returned to near baseline by 4 h. Given the robust increase in plasma corticosterone levels during the initial hours post-injection, we chose to analyze gene expression 2 h post-injection in subsequent experiments, a time point temporally consistent with direct glucocorticoid-based transcriptional mechanisms. A replicate experiment yielded a corticosterone elevation of similar magnitude, and samples from this second experiment were used for subsequent qPCR experiments (*n* = 8 animals/group, Figure 2B).

### Single injection of corticosterone results in mRNA regulation in brain *in vivo* of mRNAs previously reported to be regulated by glucocorticoids in astrocytes *in vitro*

**Figure 3:**
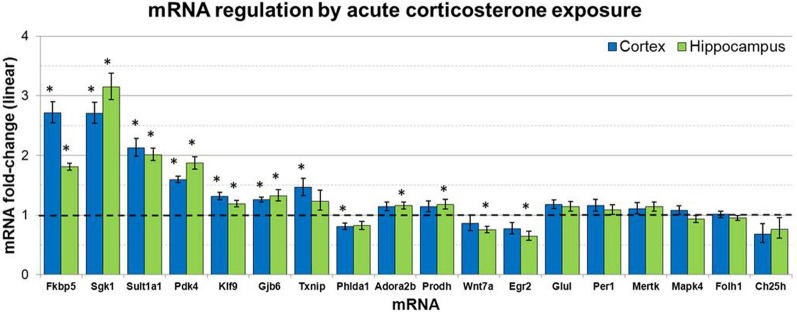
**Acute corticosterone exposure regulates select mRNAs in hippocampus and prefrontal cortex.** Gene expression fold-change ratios for mRNAs regulated by glucocorticoids in astrocytes *in vitro* (Carter et al., 2012) in prefrontal cortex and hippocampus following injection of 10 mg/kg corticosterone vs. saline control *in vivo* (2 h post-injection exposure, *n* 8 per group). Dashed line indicates no change compared to vehicle. Error bars = SEM. ^*^p < 0.05.

mRNA levels for select genes in RNA samples derived from two brain regions (prefrontal cortex and hippocampus) of mice injected with corticosterone or vehicle solution were measured by qPCR using Taqman Low Density Arrays (TLDAs). 18 mRNAs were measured based on robust glucocorticoid-sensitivity in astrocytes *in vitro* (Carter et al., 2012). Twelve of these 18 mRNAs were statistically regulated by acute corticosterone exposure *in vivo* in at least one of the brain regions tested (Figure 3). Among all mRNAs measured, 6 mRNAs were differentially expressed in both cortex and hippocampus following acute corticosterone treatment (upregulated; Fkbp5, Gjb6, Klf9, Pdk4, Sgk1, Sult1a1). 2 mRNAs were regulated in cortex but not in hippocampus (upregulated: Txnip; downregulated: Phlda1), and 6 mRNAs were regulated in hippocampus but not cortex (upregulated: Adora2b, Egr2; downregulated: Hdac7, Prodh, Slc1a2, Wnt7a). 15 mRNAs that have been reported to be regulated by glucocorticoids *in vitro* [listed in Carter et al. (2012)] were not statistically regulated in either brain region by acute corticosterone exposure *in vivo* (previously reported upregulation: Ch25h, Folh1, Foxo1, Gap43, Glul, Mapk4, Mertk, Per1, Syn2; previously reported downregulation: Atp6v1b2, Gfap). 3 mRNAs were regulated by acute corticosterone treatment *in vivo* that were not regulated or regulated in the opposite direction by glucocorticoids in astrocytes *in vitro* (downregulated: Hdac7, Slc1a2; downregulated instead of upregulated: Egr2). The magnitude of regulation induced by acute corticosterone exposure in this condition ranged from +3.15-fold regulation (Sgk1 in hippocampus) to +0.65/−1.55-fold regulation (Egr2 in hippocampus). Complete numerical values of mRNA regulation by acute corticosterone exposure *in vivo* and corresponding statistical *p*-values for all mRNAs measured are listed in Table 3.

**Table 3:**
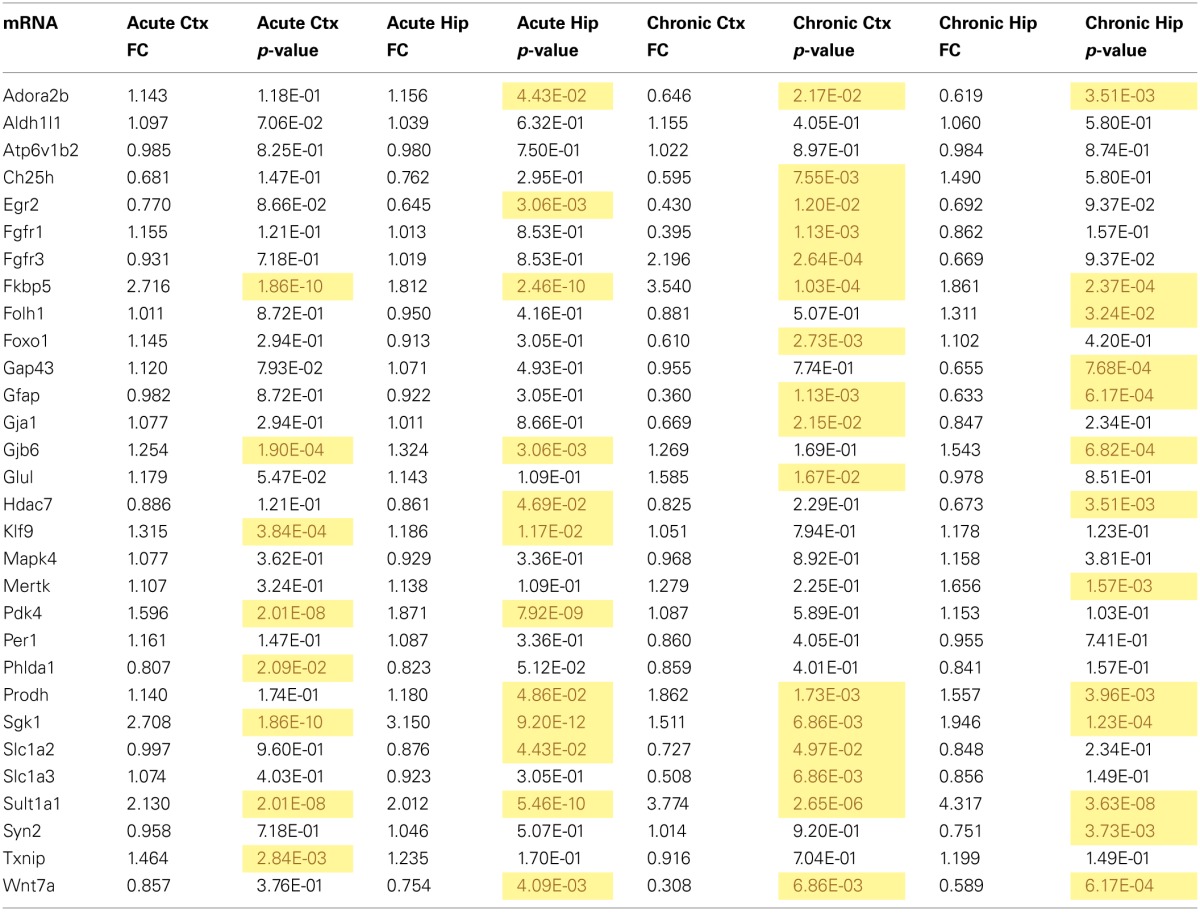
**qPCR data for all mRNAs measured for mRNA regulation by acute and chronic corticosterone exposure in the brain *in vivo***.

Ctx, cortex; Hip, hippocampus; yellow, p < 0.05.

### Mice given extended access to drinking water containing corticosterone demonstrate hormonal and organ changes consistent with acute and chronic glucocorticoid elevation

**Figure 4:**
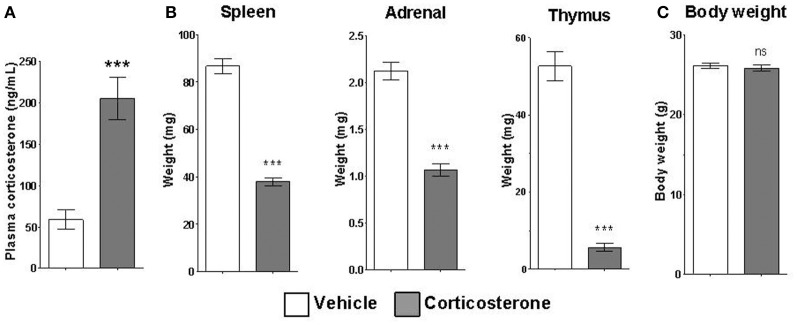
**Chronic corticosterone administration via drinking water results in acute and chronic corticosterone elevations. (A)** Plasma corticosterone levels after 2 weeks access to drinking water containing 10 mg/mL corticosterone vs. vehicle. **(B)** Weights of corticosterone-sensitive organs after chronic corticosterone administration. **(C)** Body weight of mice after chronic corticosterone administration. *N* = 5 per group. Error bars = SEM. ^***^p < 0.0001. *ns* = not significant.

Mice given access to drinking water containing corticosterone for 14 days displayed statistically elevated corticosterone plasma levels relative to mice given access to vehicle drinking water (Figure 4A). Chronic corticosterone-treated mice also had significantly reduced thymus, adrenal, and spleen organ weights compared to vehicle-treated mice (Figure 4B), data consistent with chronic glucocorticoid exposure. There was no difference in the total body weight between treatment groups (Figure 4C).

### Chronic corticosterone exposure results in mRNA regulation in brain *in vivo* of mRNAs previously reported to be regulated by glucocorticoids in astrocytes *in vitro*

**Figure 5:**
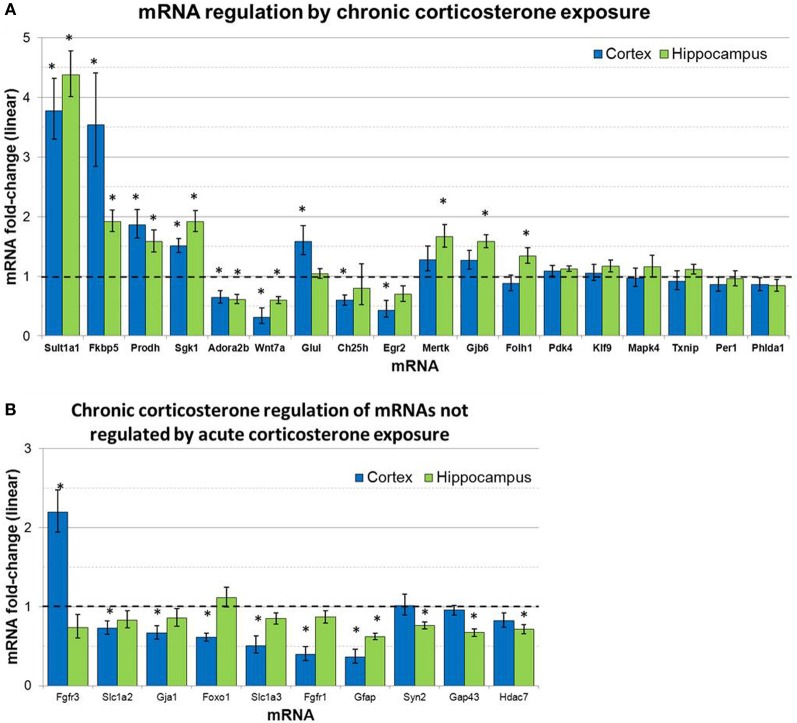
**Chronic corticosterone exposure regulates select mRNAs in hippocampus and prefrontal cortex. (A)** Gene expression fold-change ratios for mRNAs regulated by glucocorticoids in astrocytes *in vitro* (Carter et al., 2012) in prefrontal cortex and hippocampus following chronic corticosterone exposure for 2 weeks vs. vehicle control (*n* = 8 per group). **(B)** mRNA fold-changes of mRNAs regulated by chronic corticosterone exposure *in vivo* that were not regulated by glucocorticoids in astrocytes *in vitro* or acute corticosterone exposure *in vivo*. Dashed line indicates no change compared to vehicle. Error bars = SEM. ^*^p < 0.05.

mRNA levels for select genes in RNA samples derived from two brain regions (prefrontal cortex and hippocampus) of mice treated with chronic corticosterone solution or vehicle solution were measured by qPCR using TLDAs. Of the 18 mRNAs measured that were regulated by glucocorticoids in astrocytes *in vitro* (Carter et al., 2012), 12 of these mRNAs were statistically regulated by chronic corticosterone exposure *in vivo* in at least one of the brain regions tested (Figure 5). Among all mRNA measured, 7 mRNAs were regulated by chronic corticosterone exposure in both cortex and hippocampus (upregulated: Adora2b, Fkbp5, Prodh, Sgk1, Sult1a1; downregulated: Gfap, Wnt7a). 9 mRNAs were regulated by chronic corticosterone exposure in cortex but not in hippocampus (upregulated: Ch25h, Egr2, Fgfr3, Foxo1, Glul; downregulated: Ch25h, Fgfr3, Gja1, Slc1a2, Slc1a3); 6 mRNAs were regulated by chronic corticosterone exposure in hippocampus but not cortex (upregulated: Folh1, Gap43, Gjb6, Mertk, Syn2; downregulated: Hdac7). 7 mRNAs that have been reported to be regulated *in vitro* were not regulated in either region by chronic corticosterone exposure *in vivo* [previously reported upregulation: Klf9, Mapk4, Per1, Pdk4, Txnip; previously reported downregulation: Atp6v1b2, Phlda1; referenced in (Carter et al., 2012)]. 15 mRNAs were regulated in at least one brain region in a manner consistent with previously reported glucocorticoid regulation *in vitro* [listed in Carter et al. (2012)] (upregulated: Adora2b, Fkbp5, Folh1, Gap43, Gjb6, Glul, Mertk, Prodh, Sgk1, Sult1a1, Syn2; downregulated: Ch25h, Gfap, Gja1, Wnt7a) (Figure 5A). In addition, 12 mRNAs were regulated by chronic corticosterone treatment *in vivo* that were not regulated or regulated in the opposite direction by glucocorticoids in astrocytes *in vitro* (upregulated: Fgfr3; downregulated: Fgfr1, Foxo1, Gap43, Gfap, Gja1, Hdac7, Slc1a2, Slc1a3, Syn2; downregulated instead of upregulated: Egr2, Adora2b) (Figure 5B). The magnitude of regulation induced by chronic corticosterone exposure in this condition ranged from +4.38-fold regulation (Sult1a1 in hippocampus) to +0.31/−3.24-fold regulation (Wnt7a in frontal cortex). Complete numerical values of mRNA regulation by chronic corticosterone exposure *in vivo* and corresponding statistical *p*-values are for all mRNAs measured are listed in Table 3.

### Select mRNAs regulated by chronic corticosterone exposure *in vivo* have neuroanatomical expression patterns consistent with astrocyte marker Aldh1L1

**Figure 6:**
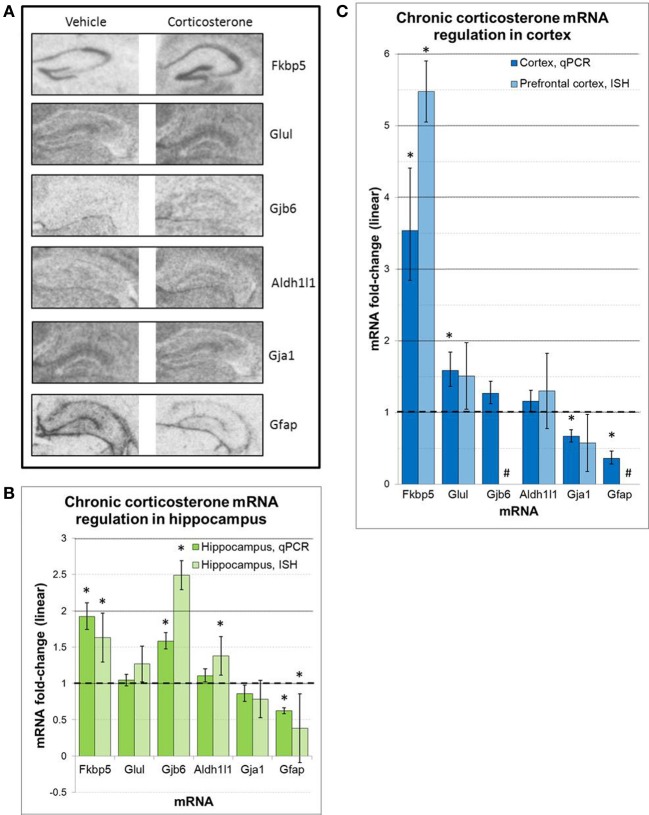
**ISH measures of select mRNAs confirm chronic corticosterone regulation and reveal mRNA expression patterns consistent with pan-astrocyte marker Aldh1l1 (A).** Representative images of *in situ* hybridization data for select mRNAs in hippocampus. **(B,C)** *in situ* hybridization densitometry measures of differential mRNA expression in whole hippocampus **(B)** and prefrontal cortex **(C)**. *n* = 5/group. Dashed line = no change compared to vehicle. ^*^p < 0.05. ^#^sub-threshold ISH signal.

Regulation of select mRNAs by chronic corticosterone exposure was further assessed in the prefrontal cortex and hippocampus by semi-quantitative radioactive *in situ* hybridization (Figure 6). These mRNAs were selected based on previously reported glucocorticoid sensitivity (Fkbp5, Gfap, Glul), their regulation by chronic corticosterone exposure in cortex and/or hippocampus based on qPCR measurements (Fkbp5, Gfap, Gja1, Gjb6, Glul), and/or an established association with astrocyte localization and function (Aldh1l1, Gfap, Gja1, Gjb6, Glul). Of the 6 mRNAs examined via *in situ* hybridization, 3 mRNAs were statistically regulated in at least one brain region in the same direction as the qPCR data (upregulated: Fkbp5, Gfap, Gjb6). 2 mRNAs demonstrated non-significant trends in the same direction as the qPCR data (upregulated: Glul; downregulated: Gja1), and 1 mRNA demonstrated differential expression in at least one brain region that was not observed by qPCR (upregulated: Aldh1l1). In terms of hippocampal expression patterns between chronic corticosterone treatment and vehicle treatment, these mRNAs showed generally consistent expression patterns between treatment groups (Figure 6A). Fkbp5 appeared predominantly expressed in pyramidal cell hippocampal subfields, while expression of Gfap, Gja1, Gjb6, and Glul was noticeably lacking in the pyramidal cell regions but displayed diffuse levels of expression in molecular cell regions. This latter anatomical distribution was highly similar to the expression pattern of the pan-astrocytic marker Aldh1l1 (Figure 6A).

### Acute corticosterone exposure *in vivo* and chronic corticosterone exposure *in vivo* (1) regulate distinct sets of mRNAs among mRNAs previously reported to be regulated by glucocorticoids in astrocytes *in vitro* and (2) demonstrate positive correlation among the expression levels of regulated mRNAs between conditions

**Figure 7:**
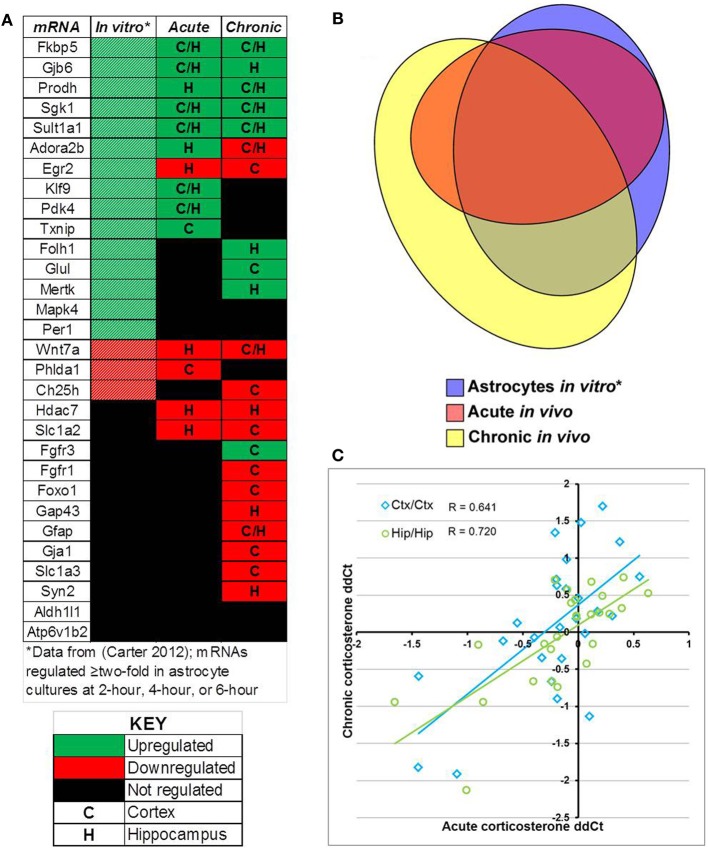
**Summary of results for measured mRNAs regarding glucocorticoid regulation by acute corticosterone exposure and chronic corticosterone exposure *in vivo*. (A)** Heatmap summarizing directionality of glucocorticoid regulation of mRNAs across conditions. Key summarizes table terminology. **(B)** Proportional Venn diagram comparing measured mRNAs regulated by glucocorticoid treatment among 3 experimental conditions (astrocytes *in vitro*, acute exposure *in vivo*, chronic exposure *in vivo*). **(C)** Plot of mRNA regulation by acute corticosterone exposure vs. mRNA regulation by chronic corticosterone exposure for all mRNAs regulated in at least one *in vivo* condition (log_2_ scale). Regulated = *p* < 0.05. ^*^in vitro data from Carter et al. (2012).

Comparison of select mRNAs measured by qPCR that were regulated by glucocorticoids in astrocytes *in vitro* [18 regulated mRNAs, (Carter et al., 2012)], acute corticosterone exposure *in vivo* (14 regulated mRNAs), or chronic corticosterone exposure *in vivo* (22 regulated mRNAs) revealed significant overlap yet also distinct patterns of mRNA regulation between conditions (*in vitro* + acute overlap: 10 mRNAs, *in vitro* + chronic overlap: 12 mRNAs, acute + chronic overlap: 10 mRNAs, Figure 7). For mRNAs regulated in multiple conditions, the directionality of glucocorticoid regulation was generally consistent across conditions (Figure 7A). 8 mRNAs were regulated in all three conditions, and all mRNAs regulated by acute corticosterone exposure *in vivo* were also regulated in one of the other conditions (Figure 7B). Among mRNAs regulated by glucocorticoids in at least one *in vivo* condition, there was a moderate, positive correlation among the measured mRNAs between mRNA expression changes between the two conditions (cortex correlation coefficient *R* = 0.641, hippocampus correlation coefficient *R* = 0.720) (Figure 7C).

## Discussion

Glucocorticoids are important mediators of the classic stress response. Previous reports have characterized extensive glucocorticoid-mediated mRNA regulation in CNS tissue using *in vitro* systems and hippocampal slice models, demonstrating that stress hormones indeed have a broad impact on mRNA expression in the brain. Although many cell types in the brain express receptors for glucocorticoids [e.g., neurons (McEwen et al., 1968), astrocytes and oligodendrocytes (Vielkind et al., 1990), microglia (Sierra et al., 2008)] and are thus presumably responsive to glucocorticoids, most studies of glucocorticoid signaling in the brain have interpreted resulting data in terms of glucocorticoid regulation in neurons. Relatively little is known about the impact of glucocorticoids on other brain cell types (e.g., glia). We and others have recently investigated glucocorticoid regulation in astrocytes *in vitro* using primary cortical astrocyte cultures derived from newborn mice and reported extensive time-dependent gene expression changes (Carter et al., 2012; Slezak et al., 2013), including glucocorticoid regulation of astrocyte-enriched mRNAs (Cahoy et al., 2008). While these *in vitro* data are intriguing, physiological significance of these results would hinge upon *in vivo* evidence of similar glucocorticoid regulation of astrocyte-enriched mRNA expression. Here we have examined the effect of exposing the brain to acute and chronic glucocorticoid treatment *in vivo* on the expression of select mRNAs that are regulated by glucocorticoids in astrocytes *in vitro*.

We first determined if a subset of mRNAs robustly regulated by short-term glucocorticoid treatment in astrocytes *in vitro* [i.e., 18 mRNAs identified in Carter et al. (2012)] were also regulated by acute glucocorticoid exposure in the brain *in vivo*. We measured mRNA expression in prefrontal cortex and hippocampus, two regions of well-characterized glucocorticoid action in the brain. A majority of the measured mRNAs regulated by glucocorticoids in astrocytes *in vitro* were also regulated by acute corticosterone exposure in at least one brain region *in vivo* (12/18 regulated, Figure 3). Almost all of the observed mRNA regulation *in vivo* was directionally consistent with the regulation in astrocytes *in vitro* (upregulated: Fkbp5, Gjb6, Prodh, Sgk1, Sult1a1, Adora2b, Klf9, Pdk4, Txnip; downregulated: Wnt7a, Phlda1). A portion of the regulated mRNAs are known to be both enriched in astrocytes and involved in specific processes, such as cellular metabolism (Pdk4, Prodh, Sult1a1), intercellular signaling (Adora2b, Gjb6, Slc1a2), and transcriptional regulation (Klf9, Txnip). While these qPCR data do not identify the cell types in which these glucocorticoid-mediated mRNA changes take place, these data confirm that these mRNAs are glucocorticoid-sensitive *in vivo*. An intriguing subsequent question will be to determine if other physiological challenges known to acutely increase glucocorticoids (e.g., stress) similarly regulate these same mRNAs. Additionally, a portion of mRNAs regulated by glucocorticoids in astrocytes *in vitro* were not regulated by acute corticosterone treatment in the brain *in vivo* (Ch25h, Folh1, Glul, Mapk4, Mertk). This result may indicate these particular mRNAs are not regulated by elevated glucocorticoids *in vivo* or that the cellular environment of the intact brain is sufficiently different than in astrocytes *in vitro* and glucocorticoid regulation of these mRNAs may be delayed and/or less robust *in vivo*. If these mRNAs are indeed regulated in the brain by glucocorticoids, another possible explanation for no observed mRNA expression changes is differential regulation by cell types; for example, glucocorticoids has been shown to increase NGF mRNA expression in neurons but decrease NGF expression in astrocytes (Lindholm et al., 1992), a scenario that may result in no net change in tissue-level mRNA expression. Alternatively, this acute corticosterone exposure condition may not be sufficient to elicit regulation of these mRNAs based on condition-dependent features (e.g., corticosterone dosage, relatively short duration of exposure). Overall, given the previous reporting of the astrocyte-enrichment of many mRNAs measured (Cahoy et al., 2008), the observed regulation of many of these mRNAs suggests that astrocytes are acutely responsive to glucocorticoids *in vivo*.

In general, the absolute magnitude of the glucocorticoid-mediated changes observed *in vivo* was lower than mRNA expression differences observed following similar steroid treatment durations in astrocytes *in vitro*. One potential explanation for this difference may be due to the active clearance of glucocorticoids from the brain compared to cell culture. In cell culture, the glucocorticoid concentration remains high and relatively constant, whereas there is a more temporally limited elevation of corticosterone in the intact brain, likely resulting in less robust regulation. Consistent with this possibility, active clearance of plasma corticosterone was directly observed in the acute exposure condition, returning corticosterone concentrations to baseline within 4 h (Figure 2A). In general, the cellular environment in the brain constantly strives to maintain homeostasis; in a situation of elevated glucocorticoid levels, the brain would engage systems to limit responsiveness to continued steroid exposure by activating opposing regulatory mechanisms to control the response to changes induced by glucocorticoid signaling. There are also less intercellular interactions in the isolated astrocyte cell culture that may counteract or limit cell-type dependent glucocorticoid responses *in vivo*. Another possible mechanism influencing the observed glucocorticoid regulation is steroid metabolism. Neurons and glia contain enzymes that modify glucocorticoid structure (e.g., 5-alpha-reductase) (Melcangi et al., 1993). The models used in these experiments cannot distinguish between regulation by corticosterone and associated metabolites in these experiments; whether directly or indirectly, we can conclude that these mRNAs are responsive to corticosterone treatment, but changes in steroid metabolism may impact the magnitude of regulation [e.g., corticosterone metabolites have different affinities for glucocorticoid receptor (McInnes et al., 2004)]. The role of glucocorticoid metabolites in the regulation of these mRNAs in the brain *in vivo* remains unresolved, which may be as or more important as regulation by the natural ligand corticosterone. Future studies that specifically utilize glucocorticoid metabolites and/or include the use of glucocorticoid receptor antagonists (e.g., RU—486) would aid in addressing this important issue. Additionally, the previous *in vitro* astrocyte experiments were focused on glucocorticoid receptor-mediated mRNA regulation; given the presence of mineralocorticoid receptor in astrocytes and the limbic system, differential receptor binding may also influence the observed mRNA regulation. Taken together, although the magnitude of mRNA regulation was reduced *in vivo* relative to astrocytes *in vitro*, these data indicate that these mRNAs can indeed be regulated in the brain by acute glucocorticoid exposure *in vivo*.

Although these data involving acute glucocorticoid regulation are relevant for understanding physiological glucocorticoid signaling (e.g., stress response), clinical conditions involving alterations in glucocorticoid signaling are often chronic in nature. Given that our acute condition resulted in a temporary increase in glucocorticoid levels, we wanted to further investigate regulation of these mRNAs in response to chronically elevated glucocorticoid levels in the brain *in vivo*. We assessed the impact of chronic glucocorticoid exposure on mice using an established protocol for administering corticosterone via drinking water (Gourley et al., 2008; Karatsoreos et al., 2010) (Figure 4). Consistent with prior studies, 2 weeks of corticosterone treatment of mice lead to elevated plasma levels of corticosterone, indicating that all mice exposed to the corticosterone solution had consumed water containing corticosterone at least in the short term. Physiological indices of chronic corticosterone treatment were observed in the form of hypotrophy of several glucocorticoid-sensitive tissues (i.e., adrenal glands, thymus, and spleen). These indices were observed without changes in body weight, suggesting limited secondary metabolic impact of this protocol. Together, these data demonstrate that mice under this protocol show signs of acute and chronic corticosterone exposure.

With evidence of chronic corticosterone exposure, we then assessed glucocorticoid-mediated mRNA regulation in the brain. Among the mRNAs measured, the majority of the mRNAs regulated by glucocorticoids in astrocytes *in vitro* were also statistically regulated in at least one brain region by chronic corticosterone exposure *in vivo* (12/18 mRNAs, Figure 5). Many mRNAs were regulated by chronic corticosterone exposure in the same direction as the glucocorticoid regulation observed in astrocytes *in vitro* and acute corticosterone exposure *in vivo* (upregulated: Fkbp5, Folh1, Gjb6, Glul, Mertk, Prodh, Sgk1, Sult1a1; downregulated: Ch25h, Hdac7, Slc1a2, Wnt7a), displaying a consistent response to glucocorticoids independent of steroid treatment duration. In contrast, several other mRNAs in the chronic corticosterone experiment were either not regulated (Klf9, Pdk4, Txnip, Mapk4, Per1, Phlda1) or regulated in the opposite direction (Adora2b, Egr2) when compared with glucocorticoid regulation observed in acute corticosterone exposure *in vivo* and astrocyte cultures *in vitro*. These data may be a result of compensatory mechanisms that reverse or limit regulation of these mRNAs under chronic glucocorticoid stimulation. Unexpectedly, chronic corticosterone exposure *in vivo* also regulated mRNAs that were not regulated in either acute glucocorticoid exposure condition *in vivo* or *in vitro* (upregulated: Fgfr3; downregulated: Fgfr1, Foxo1, Gap43, Gfap, Gja1, Slc1a3, Syn2). These discrepancies may be due to indirect, compensatory or adaptive regulation to prolonged glucocorticoid exposure. In addition, since we assessed a single chronic corticosterone exposure condition, one caveat linked to interpreting these results is that we cannot determine if the mRNAs measured change in other contexts of chronic corticosterone manipulation (e.g., longer exposures, adrenalectomy/corticosterone replacement, chronic stress). We also do not know if these changes are transient (e.g., reversible with washout period), although a previous study found long-lasting behavioral effects of chronic corticosterone exposure administered via drinking water even after an abstinence period (Gourley et al., 2008). Overall, these data provide initial evidence that astrocytes respond to chronic elevations of glucocorticoids *in vivo* and potentially demonstrate secondary regulation compared with acute glucocorticoid exposure.

A summary of the results comparing glucocorticoid regulation based on glucocorticoid exposure in astrocytes *in vitro*, acute corticosterone exposure in the brain *in vivo*, and chronic corticosterone exposure in the brain *in vivo* are shown in Figure 7. The high concordance of *in vivo* regulation by corticosterone with glucocorticoid regulation in astrocytes *in vitro* is important in part because most of these mRNAs have not been reported as glucocorticoid-sensitive in the brain *in vivo*. A subset of measured mRNAs have been reported to be regulated in the brain *in vivo* by glucocorticoids or stress conditions [e.g., Fkbp5 (Scharf et al., 2011), Gfap (O'Callaghan et al., 1989), Glul (Patel et al., 1983), Sgk1 (Sarabdjitsingh et al., 2010)], but a number of astrocyte-enriched mRNAs demonstrate novel *in vivo* glucocorticoid regulation in the brain (e.g., Gjb6, Prodh, Adora2b, Klf9, Pdk4, Txnip, Folh1, Mertk, Wnt7a). Of the mRNAs with reported glucocorticoid regulation in the brain *in vivo*, the directionality matches the previous data, giving further confidence that our data documents authentic glucocorticoid regulation in the brain. The additional *in vivo* regulation observed in these experiments may not have been previously observed for multiple reasons; one possibility is that the magnitude of changes are small enough that they may not have been statistically significant if measured with techniques less sensitive than qPCR (e.g., microarray).

In addition, these results have implications for understanding glucocorticoid regulation in astrocytes by brain region. There is increasing evidence of astrocyte diversity based on neuroanatomical location; given the extensive differential mRNA regulation among neurons, astrocytes may also exhibit differential mRNA regulation by brain region. This point is also relevant given the data comparisons to cortical astrocyte cultures *in vitro* (Carter et al., 2012). Our results characterize glucocorticoid-mediated mRNA regulation in two brain regions known to be involved in glucocorticoid action, the prefrontal cortex and hippocampus; whether these mRNAs are regulated in a similar manner in other brain regions remains to be investigated. In response to acute or chronic corticosterone exposure, certain mRNAs were statistically regulated in one brain region but not the other (e.g., Hdac7 only in hippocampus). While there may indeed be differential glucocorticoid mRNA regulation by brain region, such results may be due to specific factors unique to these particular experimental conditions (e.g., exposure times, criteria used to define differential expression). Additional experiments showing consistent brain-region-dependent glucocorticoid regulation across multiple exposure times and conditions *in vivo* are needed to address this possibility.

We also noted a moderate positive correlation between the measured mRNA changes induced by acute corticosterone exposure and chronic corticosterone exposure by brain region (Acute-Cortex vs. Chronic-Cortex: *r* = 0.641, Acute-Hippocampus vs. Chronic-Hippocampus: *r* = 0.720). In other words, mRNAs regulated by glucocorticoids in one condition generally were regulated in the other condition with a similar directionality and magnitude. Given that the mRNAs in this study were selected based on their association with glucocorticoid regulation in astrocytes, perhaps this pattern would be applicable to other glucocorticoid-regulated mRNAs in astrocytes. Although we cannot determine with these data if the observed changes in the two conditions are occurring by similar mechanisms (e.g., direct regulation by glucocorticoid receptor), these correlations suggest that robust glucocorticoid sensitivity may be able to induce regulation of select mRNAs in the brain independent of exposure duration.

Although we have substantiated the notion of glucocorticoid regulation *in vivo* of mRNAs regulated by glucocorticoids in astrocytes *in vitro* via qPCR, we cannot localize mRNA regulation to specific cell types in a multicellular brain tissue sample using this technique. In order to examine the neuroanatomical nature of these glucocorticoid-regulated mRNAs *in vivo*, we performed *in situ* hybridization studies on brain tissue from the chronic corticosterone exposure experiments (e.g., mice chronically exposed to corticosterone solution or vehicle solution). Using semi-quantitative densitometry, we were able to assess the glucocorticoid regulation of a number of mRNAs in the prefrontal cortex and/or hippocampus by ISH (Figure 6). Although only a subset of differential expression as measured by qPCR were validated by ISH, the ISH data was directionally consistent with the qPCR data in almost all cases; the discrepancy of statistical significance may be in part due to a more robust sensitivity of qPCR measurements.

The hippocampus has a well-known cytoarchitecture that can readily associate cell types with specific locations in this brain region. Within the hippocampus, there are well-known layers such as the pyramidal cell layer in the hippocampal subfields (e.g., dentate gyrus, CA1, CA2, CA3), which is characterized by a higher density of neurons compared with other regions. In contrast, the molecular layer contains a relatively lower neuron density. We thus examined the neuroantomical expression patterns of these 6 mRNAs in an effort to associate the observed mRNA regulation with the location of specific cell types. Similarly general expression patterns of these mRNAs were observed by ISH in sections from both vehicle-treated animals and corticosterone-treated animals, suggesting that the observed changes are likely occurring in cells that express these mRNAs under basal conditions. Among mRNAs measured, we detected ISH signal for Fkbp5 predominately in the pyramidal cell layer throughout multiple hippocampal subfields (Figure 6A). Following chronic glucocorticoids, the hybridization signal appears to rise across the pyramidal cell layer of the hippocampal subfields without any noticeable increase in signal in the molecular layer. While additional high resolution images will be necessary to fully determine in which cell types Fkbp5 is being regulated, this expression pattern suggests the mRNA regulation occurs in pyramidal neuronal cell populations. In contrast, mRNAs regulated by chronic corticosterone but not expressed in pyramidal cell regions may be regulated in other cell types (e.g., astrocytes). Strikingly, all of the other corticosterone-regulated mRNAs analyzed by ISH in this study demonstrate very low or undetectable levels of expression in pyramidal neuron subfields (Gfap, Glul, Gja1, Gjb6). The gene expression patterns of these mRNAs appear much more homogeneous across the hippocampus, an anatomical distribution that is largely consistent with the reported distribution of astrocytes in this tissue (Ogata and Kosaka, 2002; de Vivo et al., 2010). Importantly, these mRNAs also show similar expression patterns as that of Aldh1l1, a mRNA previously shown to be substantially enriched in astrocytes and described as a pan-astrocytic marker (Cahoy et al., 2008). While further studies will be required to definitively identify the cell type of this differential glucocorticoid-sensitive gene expression, given the similar general expression patterns of these mRNAs between corticosterone-treated and vehicle treated animals along with the highly similar anatomical distribution of Gja1, Gbj6, and Glu1 the pan-astrocytic marker Aldh1l1, these results are consistent with the hypothesis that these mRNAs are regulated by chronic glucocorticoid exposure in astrocytes *in vivo*.

We also note a differential regulation of astrocyte cell markers by corticosterone treatment *in vivo* that may be important for future studies of glucocorticoid signaling involving astrocytes. Gfap, a cytoskeletal filament and a marker historically associated with astrocytes in the brain, yields a different expression pattern compared with the pan-astrocytic marker Aldh1l1 and the other astrocyte-enriched mRNAs in the hippocampus (Figure 6A). Gfap mRNA is detected at relatively lower levels throughout the hippocampus but at relatively higher levels at the boundary of the structure; this boundary expression is consistent with previously reported Gfap expression in ependymal cells (i.e., pial distribution) (Liu et al., 2006). Following chronic glucocorticoid-treatment, Gfap mRNA levels decrease across all areas (ependymal cells and non-pyramidal cell areas of the hippocampus), consistent with our qPCR data and previous reports (O'Callaghan et al., 1989). Recent studies on major depression have used Gfap in human postmortem experiments and animal models of depression to measure astrocyte cell number; multiple reports have reported decreased astrocyte cell density in these conditions based on Gfap protein measurements (e.g., Rajkowska et al., 1999; Miguel-Hidalgo et al., 2000). However, this interpretation may be confounded by glucocorticoid-induced downregulation of Gfap mRNA and corresponding protein in these conditions associated with hypercortisolemia. If astrocyte cell numbers are changing, then all astrocyte markers would also be expected to change in parallel. We have measured Aldh1l1, a more recently identified pan-astrocyte marker, and found that Aldh1l1 is not downregulated by glucocorticoids under any measured condition (in contrast, our ISH data suggest possible upregulation by chronic corticosterone exposure). These findings suggest that astrocyte numbers may not be decreasing but that the astrocyte cytoskeleton is dynamically regulated in conditions of hypercortisolemia. Additional cell counting studies using Gfap and Aldh1l1 are needed to clarify this issue. In general, future studies in astrocytes would benefit from using multiple cell markers or a marker known not to be affected by any component of the manipulation or disease of interest.

What are the functional consequences of glucocorticoid regulation in astrocytes *in vivo*? We can project functional alterations based on the known roles of the mRNAs measured. Two potential functional alterations involve gap junction coupling and glutamate signaling. Astrocytes express two main gap junction proteins, connexin-30 (Gjb6) and connexin-43 (Gja1). Gjb6 was strongly upregulated by both acute and chronic corticosterone exposure *in vivo*, while Gja1 was downregulated by chronic corticosterone exposure. Changes in these gap junctions would likely alter the permeability within astrocytes and between astrocytes and other cells [e.g., oligodendrocytes (Orthmann-Murphy et al., 2007)], a significant alteration given the important role of gap junctions in intercellular communication among astrocytes. Astrocytes also play an important role in glutamate signaling, both in terms of glutamate synthesis and glutamate transport. While glucocorticoids have been known to increase glutamine synthetase (Glul) expression, we observed that chronic glucocorticoid exposure decreased the expression of both astrocytic glutamate transporters, GLT-1 (Slc1a2) and GLAST (Slc1a3). Decreases in astrocyte glutamate uptake have been reported in chronic stress conditions (Almeida et al., 2010; de Vasconcellos-Bittencourt et al., 2011); our results suggest that impairment of glutamate transport could be mediated by chronic glucocorticoid exposure, a potential mechanism for the changes seen in chronic stress.

These data also have interesting parallels to clinical findings in postmortem studies of major depression, a condition associated with elevations of glucocorticoids. Multiple mRNAs regulated by chronic corticosterone exposure in this study are reported to be changed in the same direction in human depression (e.g., downregulation of Gja1, Slc1a2, and Slc1a3) (Bernard et al., 2010). While the data presented in these studies cannot be directly linked to depression biology, they do provide a potential mechanism (glucocorticoid regulation) by which these genes may be altered. Alterations of astrocyte-enriched fibroblast growth factor (FGF) receptors observed here (upregulation of Fgfr3, downregulation of Fgfr1) may also be relevant to depression biology given the findings of altered expression of FGF receptors and ligands in depression (Evans et al., 2004). We would be interested in determining if these same mRNAs are regulated in other animal models demonstrating chronically elevated corticosterone levels, such as animal models of depression. Given the associations of increased depressive-like behavior and astrocyte dysfunction (Banasr and Duman, 2008), perhaps these mRNAs are relevant to the underlying mechanisms of these observations. A caveat of such extrapolations is that we have documented glucocorticoid-mediated mRNA changes but not changes in corresponding proteins; further studies are needed to measure correlations between glucocorticoid-mediated changes in mRNA expression and any changes in corresponding protein levels.

In summary, our data demonstrate that select mRNAs regulated by glucocorticoids in astrocytes *in vitro* are also regulated by acute and/or chronic corticosterone exposure *in vivo*. A number of these mRNAs have been reported as astrocyte-enriched (Cahoy et al., 2008), and ISH data of anatomical expression patterns within the hippocampus suggest that certain mRNA changes occur within regions containing astrocytes. Together, these data suggest that the observed glucocorticoid-mediated mRNA regulation may be occurring in astrocytes. These data add physiological significance to parallel gene expression findings in astrocyte cell culture and suggest that additional findings of glucocorticoid regulation in astrocyte cultures *in vitro* may extend to the brain *in vivo*. Based on these results, we posit that differential responses to glucocorticoids by cell type is an important factor for fully understanding glucocorticoid-mediated mechanisms in the brain. The functional contribution of glucocorticoid regulation in astrocytes to stress signaling remains to be investigated and may be integral to both the classical stress response and pathological conditions associated with elevations in glucocorticoid levels in the brain.

### Conflict of interest statement

The authors declare that the research was conducted in the absence of any commercial or financial relationships that could be construed as a potential conflict of interest.
